# Quad-band SIW antenna with micro-pocket enabled frequency-agile design for 5G/6G IoT applications

**DOI:** 10.1038/s41598-026-46067-y

**Published:** 2026-03-28

**Authors:** P. Vaishali, Sounik Kiran Kumar Dash, Rusan Kumar Barik, Mohammad Junaid Khan, Niraj Kumar Dewangan

**Affiliations:** 1 Department of Electronics and Communication Engineering, SRM Institute of Science and Technology, Kattankulathur, Tamil Nadu 603203 India; 2 Department of Electronics and Communication Engineering, School of Engineering and Technology, CHRIST University, Bangalore, India; 3 Department of Electronics and Communication Engineering, SR University, Warangal, Telangana India; 4 Manipal Institute of Technology, Manipal Academy of Higher Education, Manipal, India

**Keywords:** Antenna design, 5G/6G, IOT applications, SIW, Engineering, Physics

## Abstract

A single polarized substrate integrated waveguide (SIW) cavity supported self-quadruplexing antenna, designed for 5G/6G IoT applications is proposed and prototyped. The model is backed by a rectangular substrate integrated waveguide (RSIW) cavity and features four resonating patches excited separately through four different 50Ω feed lines. The antenna center frequencies are obtained at 3.29 GHz, 4.47 GHz, 5.85 GHz, and 7.07 GHz. Additionally, the cavity is engineered with four sets of micro pockets beneath the patches which can be filled with different materials to offer frequency-agile response. The operating frequencies can be tuned over a wide range between 3.29 GHz and 8.4 GHz as per the required targets. The layout of the model is chosen meticulously to ensure all ports are co-polarized and isolation between any two is better than 32 dB. The proposed antenna design exhibits competitive performances with a compact size of 0.09 *λ*_*g*_², Front-To-Back-Ratio (FTBR) above 17.83 dB and peak gain of 7.6 dBi. Importantly, all ports are single polarized for the first time in their class. The performance is validated by an equivalent circuit model and prototype characterization. The proposed antenna specifications and configurations well suit for future high-end applications like IoT/5G/6G/satellite communications.

## Introduction

**Fig. 1:**
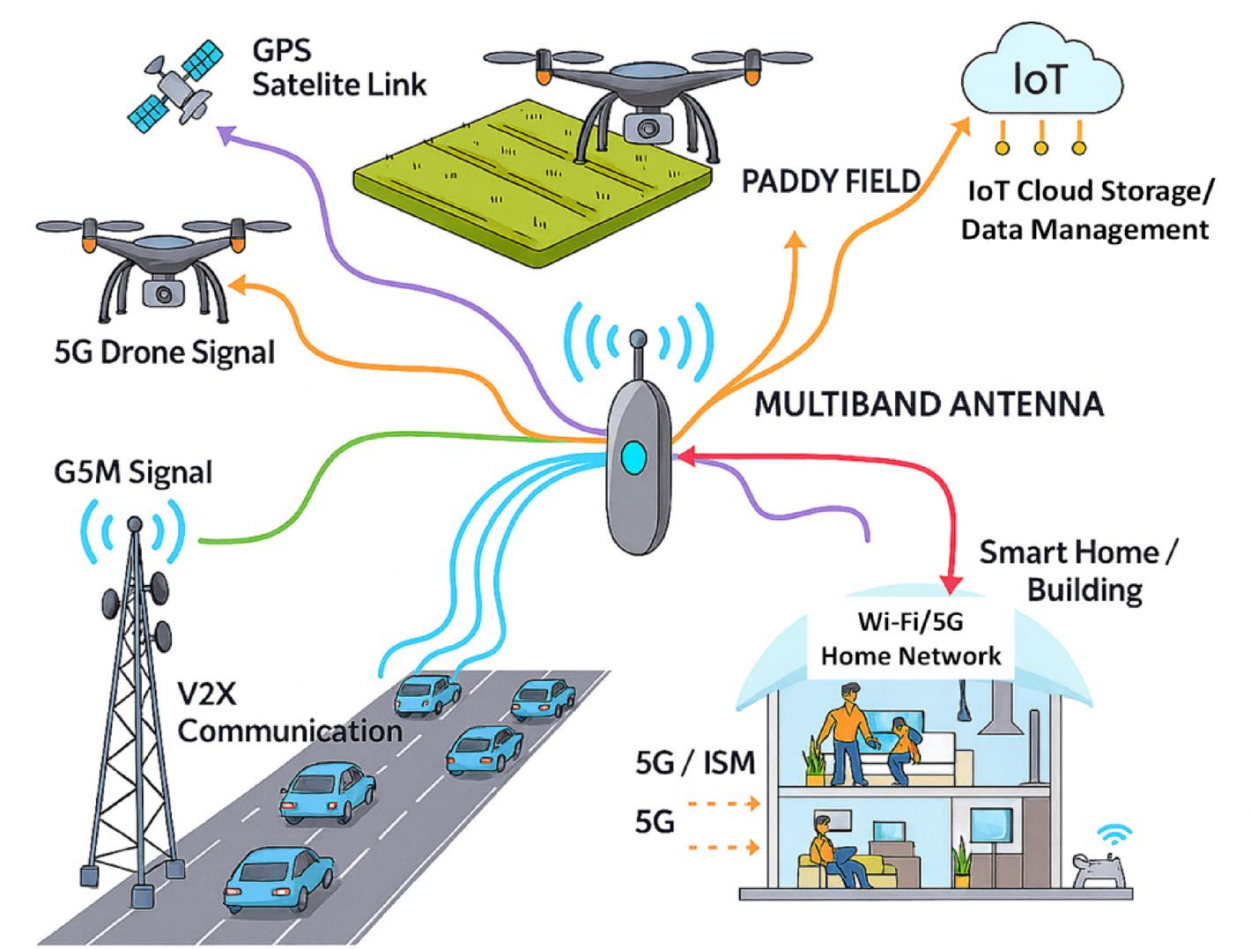
A representative image illustrating the cutting-edge applications of multiband antennas.

The Modern wireless communication systems have driven the need for antennas that support high data rates, multi-channel communication, and compact structure^1^. Multi-band antennas have emerged as a guaranteed solution for addressing these requirements^2^ as demonstrated in Fig. 1. However, using multiband antennas is challenging, as they require an additional circuitry, such as multiplexer to manage signal transmission and reception^3^, thereby increasing the total device size and making them not ideal for compact handheld devices.

Self-multiplexing antennas (SMA) can handle multiple signals within a single structure for both transmission and reception. This eliminates the need for an external decoupling network, such as the multiplexer circuit used in conventional multiband antennas, and minimizes the system size since no active elements are required^4^. Such antennas are termed as self-diplexing, self-triplexing, self-quadruplexing, corresponding to layouts having dual, triple, and quad ports, respectively.

Substrate-integrated waveguide (SIW) technology is an effective approach for implementing compact, efficient, and low-loss RF systems^5^. It provides unique features such as high directivity, high front-to-back-ratio (FTBR), good isolation, and excellent electromagnetic (EM) energy confinement, making it an excellent option for backing multi-band antennas. Integrating SIW with self-multiplexing antennas creates high-performance devices with a simple and compact structure and it makes integration with planar devices easier due to its high isolation properties^6^.

The concept of self-diplexing was first introduced with high isolation by minimizing the active components and overall weight of the diplexer structure^7^. Later, the combination of SMA’s with SIW technology was adopted to further enhance performance. Several techniques have been reported for integrating self-multiplexing antennas with SIW structures, leading to successful implementations of dual-band^1–11^, triple-band^12–16^, and quad-band antennas^17–27^ using SIW cavities for various applications.

Some works include: self-diplexing antennas employing SIW cavities with bowtie-shaped slots^1^, two transverse slots^2^, plus-shaped slots^3^, bowtie shaped slots^4^, dual-cavity^5^, dual rectangular slots^6^ and so on^8,9^. Similarly, self-triplexing antennas have been realized with dual bowtie slots^12^, a combination of one annular and two transverse slots^13^, I-shaped slots^14^ and so on^15,16^. Self-quadruplexing antennas have been demonstrated using four V-shaped slots^17^, patches of varying lengths^18^ and similar approaches^19–27^.

Some of the SIW cavity-backed triplexers, at least one operating frequency is obtained by the coaxial probe feed method^13,14^. However, the use of a coaxial probe feed limits its application in a planar device. Apart, most of the aforementioned models offer frequency tuning (restricted to pre-fabrication). Only a few studies have explored post-fabrication tuning methods, such as solid or liquid-filled micro-pockets for self-multiplexed multiband antennas^8–10,23–25,27^ and higher order MIMO antennas as well^27^. And importantly, in almost all of the above models due to their feeding mechanism the polarization of each port/band is not the same. Since these bands operate with different polarizations, these models may be challenged in the future as an alternative to conventional multiband antennas, where all bands typically exhibit uniform polarization.

Hence, developing a novel compact SMA design with high isolation, post fabrication tuning flexibility, all planar ports, and uniform polarization at all bands/ports remains a challenge. Here, a novel compact self-quadruplexing antenna (SQA) backed by a rectangular SIW (RSIW) cavity is proposed, which does not require any external multiplexing circuitry for independent band selection. The key novelties of the proposed antenna are:

i) The ports in the SQA are arranged along one axis and ensure all bands operate in uniform polarization like a multiband antenna for the first time in its class.
ii) The antenna offers a very small footprint (18.18% more compact than the most compact design^26^ reported in literature).
iii) The model is equipped with a set of micro pockets which can be filled with different materials to offer active/post fabrication frequency tuning between 3.29 and 8.4 GHz.
iv) All ports are planar and suitable for planar devices.
v) The model is validated by prototype characterization and equivalent circuit analysis.

The proposed structure, design analysis and results are discussed in the upcoming sections.

## Methods

### Antenna configuration

**Fig. 2:**
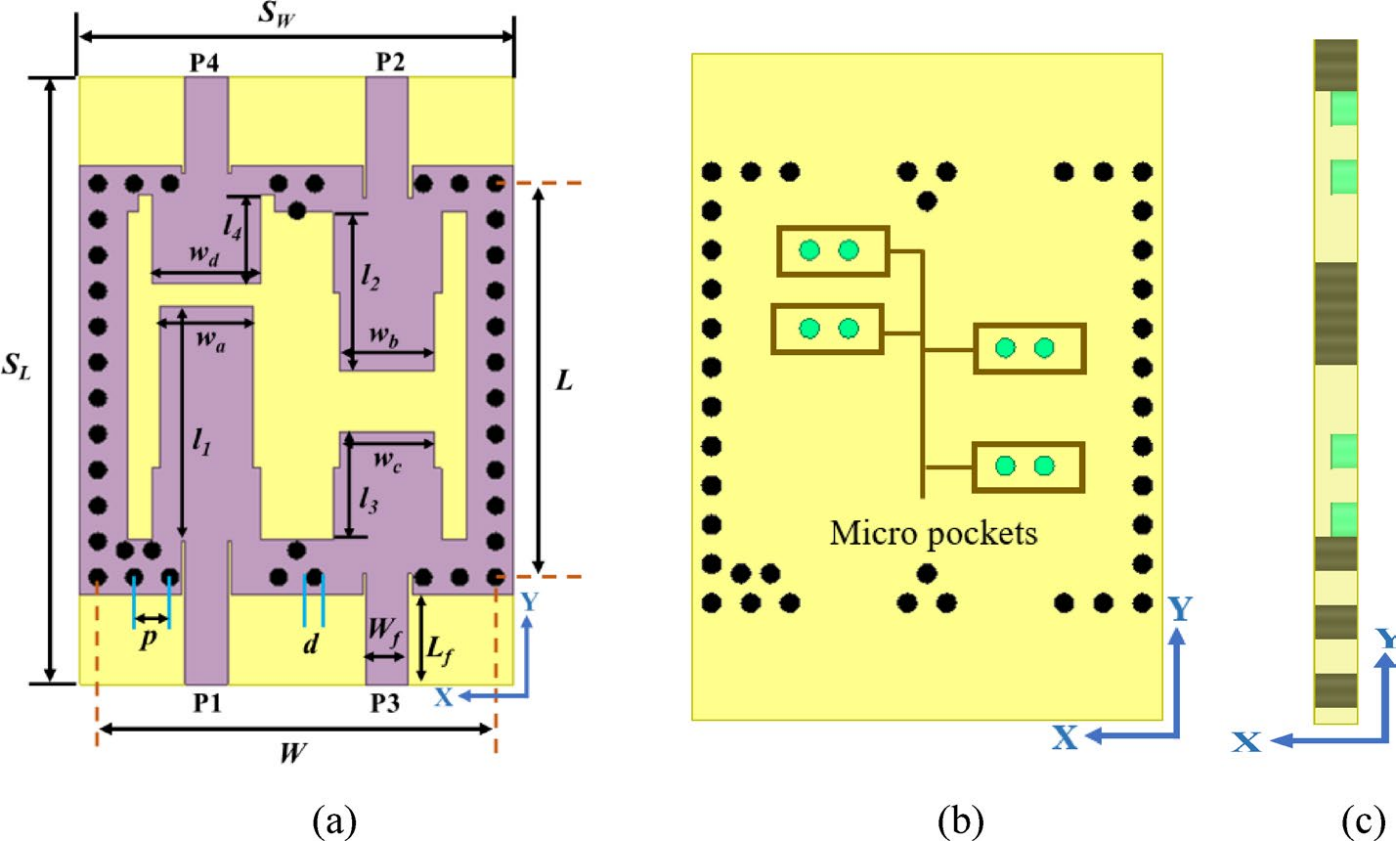
Illustration of the proposed antenna structure (**a**) top (**b**) bottom, and (**c**) side view.

The proposed RSIW backed quad band structure is constructed on Rogers RT/Duroid 5870 substrate, having a dielectric constant (*ε*_*r*_) of 2.33 and a loss tangent (*tan δ*) of 0.0012. The top of the proposed SQA is engineered into four resonators, which are fed individually by four microstrip 50Ω feedlines which makes it suitable for planar devices. The schematic view of the proposed structure is illustrated in Fig. 2 and its physical dimensions are listed in Table 1. To minimize coupling (> 20 dB) between any two ports, stepped patches and extra vias are introduced to the proposed structure.

**Fig. 3:**
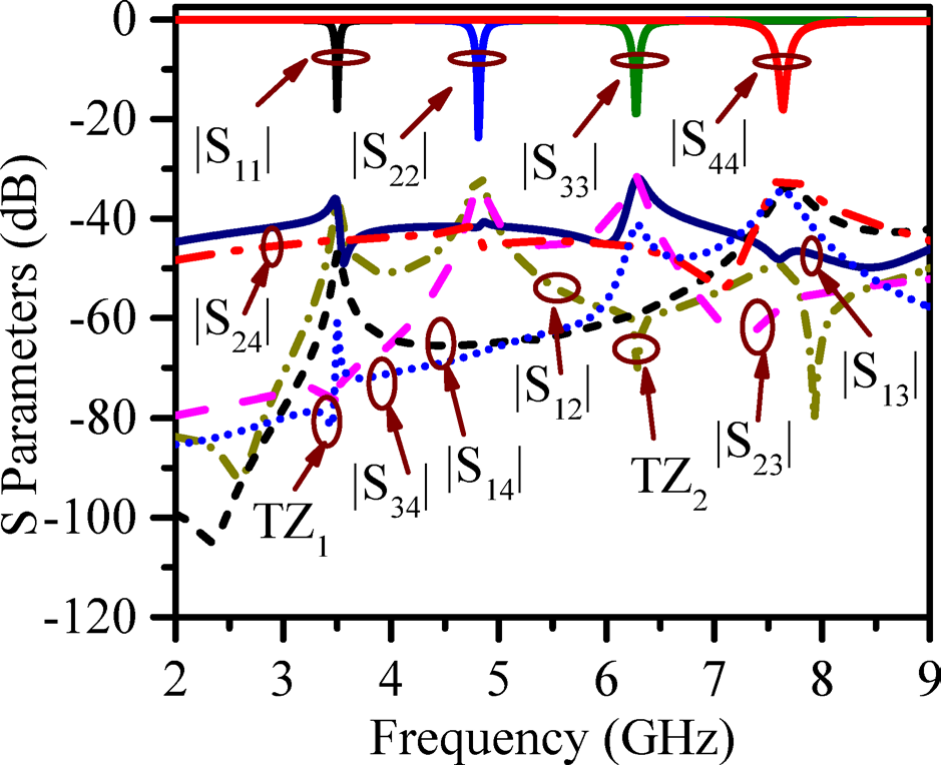
The simulated S-parameters of the proposed RSIW backed SQA model.

**Table 1:** Physical dimensions of the proposed structure.

Parameters	Values (mm)	Parameters	Values (mm)
*L*	22	*l*_1_	13.1
*W*	22	*l*_2_	8.9
*p*	2	*l*_3_	6
*d*	1	*l*_4_	5
*L*_*f*_	5	*w*_*a*_	5.2
*W*_*f*_	2.33	*w*_*b*_	5.2
*S*_*L*_	34	*w*_c_	5.2
*S*_*W*_	24	*w*_d_	6

Figure 3 shows the simulated S-parameters of the proposed RSIW backed SQA. It is seen that the frequencies of operations are 3.5 GHz, 4.81 GHz, 6.27 GHz, and 7.64 GHz. The design exhibits multiple transmission zeros (TZ₁, TZ₂), as observed in the S-parameters, ensuring isolation better than 32 dB across the entire band with minimal or negligible energy transfer between ports.

### Development stages of the antenna

The step-by-step development stages of the proposed RSIW backed SQA structure is given in Table 2.

**Table 2:** Development stages of the proposed antenna.

Stages	Description
Stage 1	A full-mode SIW cavity with feedlines is developed initially. Then the operating frequency at which the cavity resonates is calculated using the following formula^4^; ${f_{mn0}} = \;\frac{1}{{2\pi \sqrt {\mu \varepsilon } }}\sqrt {{{\left( {\frac{{m\pi }}{{{L_{eff}}}}} \right)}^2} + {{\left( {\frac{{n\pi }}{{{W_{eff}}}}} \right)}^2}} \;\;\;\;\;\;\left( 1 \right)$ ${L_{eff}} = L - 1.08\frac{{{d^2}}}{p} + 0.1\frac{{{d^2}}}{L}\;\;\;\;\;\;\;\;\;\;\;\;\;\;\;\;\;\;\;\;\;\;\left( 2 \right)$ ${W_{eff}} = W - 1.08\frac{{{d^2}}}{p} + 0.1\frac{{{d^2}}}{W}\;\;\;\;\;\;\;\;\;\;\;\;\;\;\;\;\;\;\;\left( 3 \right)$ Where *m* and *n are* 1, 2, …, ε is the relative permeability or dielectric constant of the substrate, *L*_*eff*_ and *W*_*eff*_ are the effective length and width of the cavity.To achieve minimal energy loss and dispersion, the cavity wall is constructed using chain of metallic vias of diameter (*d*) and pitch distance (*p*), where 0.5*p* ≤ *d* ≤ 0.1*λ*_*g*_ to prevent energy dispersion^16,]^^20,]^^22^. Four 50Ω feedlines are added to the structure to provide excitation.
Stage 2	A rectangular slot is constructed on the top side of the developed full-mode SIW structure. The isolation level of this structure is analysed for further development of the design.
Stage 3	Four resonating patches of different lengths (*l*_*1*_ to *l*_*4*_) are added to the structure to achieve the quad-band operation.
Stage 4	Additional vias and stepped patches are added to the antenna structure to improve isolation between the ports.
Stage 5	Micro-pockets are incorporated at the bottom of the antenna structure. These micro-pockets when filled with desired solid and liquid dielectrics of higher permittivity, enable post-fabrication tuning. A detailed discussion of this stage can be found in the post-fabrication frequency tuning analysis.

**Fig. 4:**
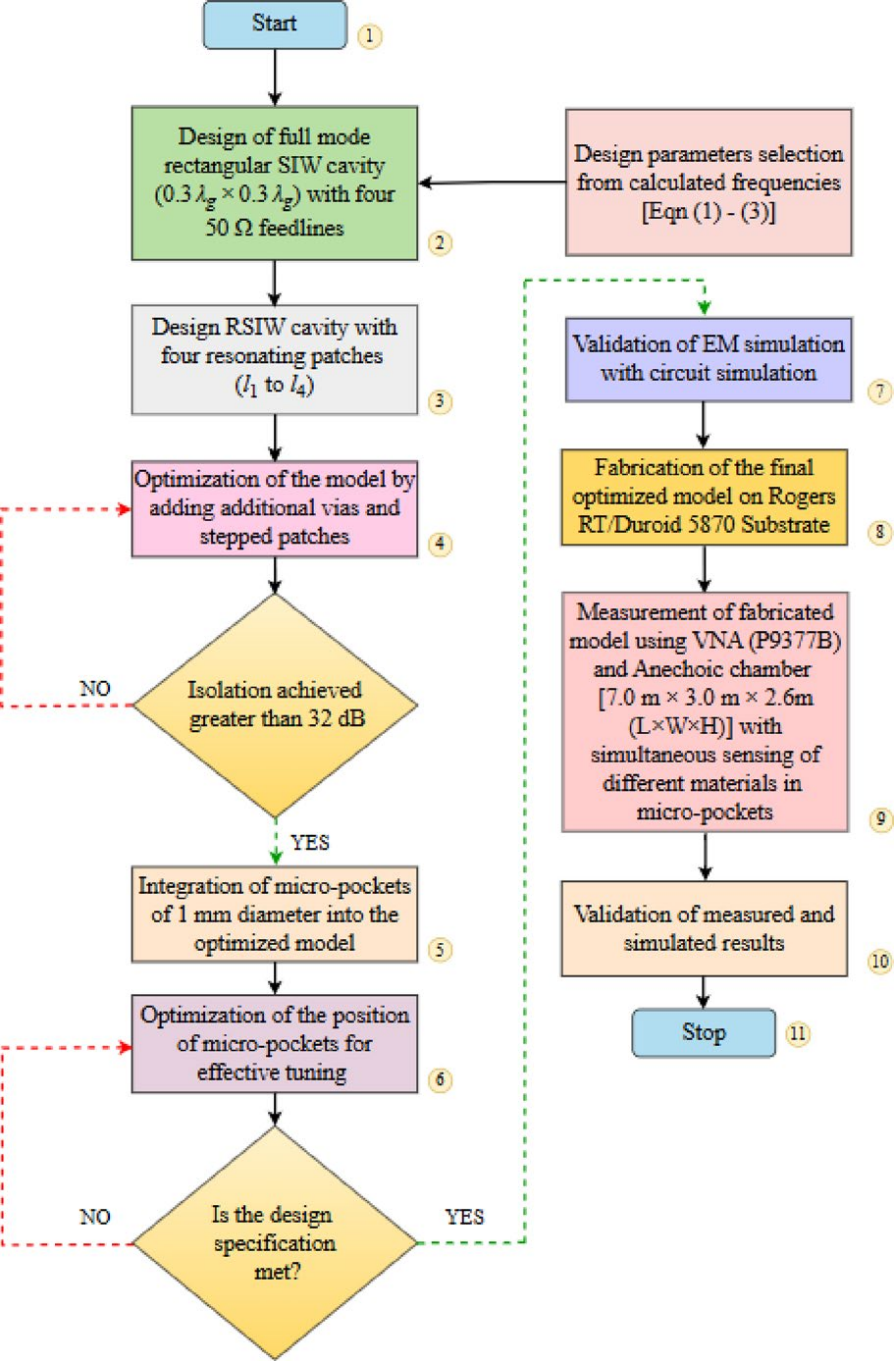
Flowchart showing development of the proposed model.

**Fig. 5:**
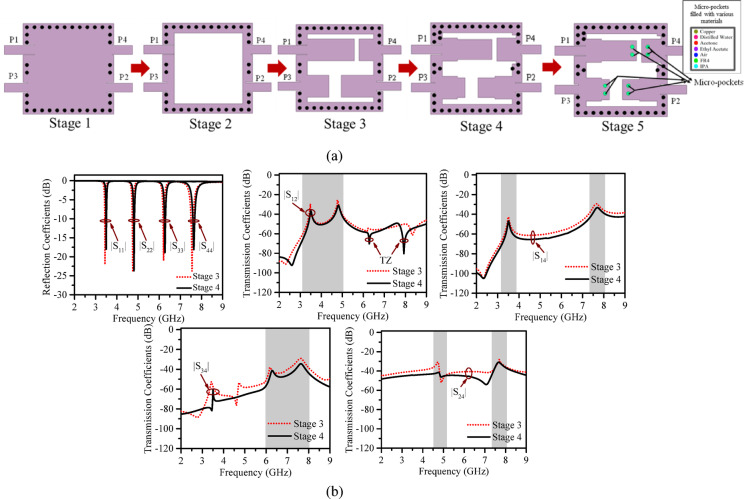
(**a**) Step by step design of the proposed structure from Stage 1 to Stage 5 (**b**) Stage 3 Vs Stage 4 - S parameter comparison.

The straightforward design guidelines of the proposed model are depicted in the flow chart in Fig. 4. The development stages of the proposed structure are illustrated in Fig. 5a. In Fig. 5b, the S parameters at stage 3 and stage 4 are compared. It is seen, placement of additional vias and stepped patches improves the isolation at all ports (minimum > 32 dB).

### Surface electric field distribution

**Fig. 6:**
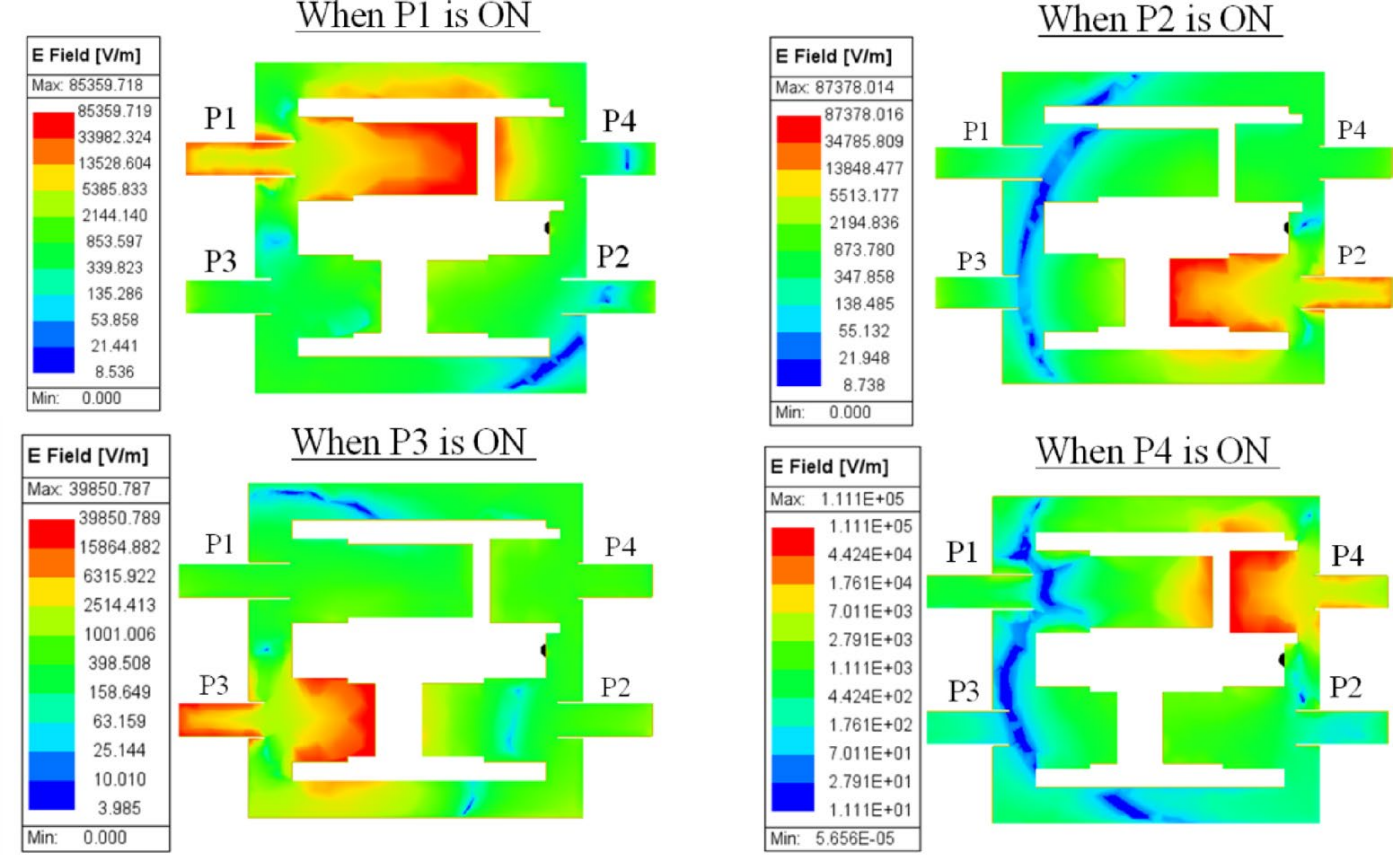
Electric Field distribution of the proposed self-quadruplexing antenna model at each port.

To understand the EM energy interaction among the patches/ports, the surface E-field distribution at various ports of the proposed antenna model is analyzed and demonstrated in Fig. 6. The corresponding E-field distribution (in V/m) for each port excitation is summarized in Table 3. It is seen that the peak E-field difference between the excited port and other non-excited ports is ~ 80%, which confirms that, when a certain port is active/high, the energy is mostly restricted to the respective patch areas (see color code in Fig. 6) with limited/minimal energy transfer to the nearby/adjacent ports and ensures strong port-to-port isolation.

**Table 3:** E-field values at excited and non-excited ports.

Excited Port	Peak E-Field at Excited Port (V/m)	Peak E-Field at Non-Excited Ports (V/m)	Difference (%)
P1	9.35 × 10⁴	≤ 1.76 × 10⁴	~ 80% lower
P2	8.67 × 10⁴	≤ 1.75 × 10⁴	~ 80% lower
P3	9.55 × 10⁴	≤ 1.70 × 10⁴	~ 82% lower
P4	1.11 × 10⁵	≤ 1.76 × 10⁴	~ 84% lower

### Frequency agile analysis

The proposed model exhibits frequency agility and supports multi-band operation thereby improving spectral efficiency. Additionally, a frequency ratio analysis has been done to determine the minimum spacing at which two resonant frequencies can occur within acceptable isolation. This helps in designing a compact and efficient antenna structure.

### Pre-fabrication frequency tuning

**Fig. 7:**
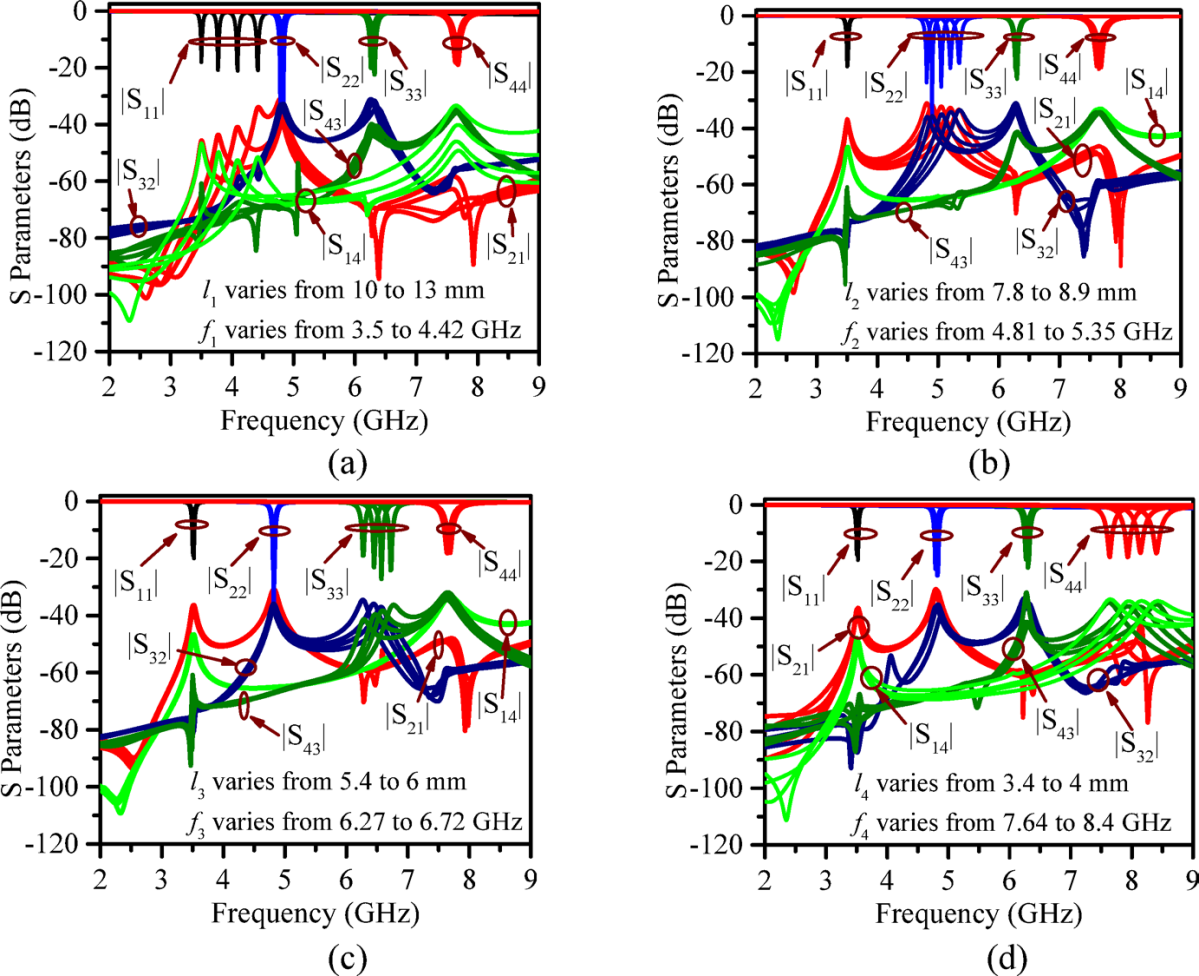
Frequency tuning by varying the length of the patch (**a**) *l*_1_, (**b**) *l*_2_, (**c**) *l*_3_, (**d**) *l*_4_ of the proposed antenna structure before fabrication.

The proposed antenna undergoes pre-fabrication tuning by varying the radiating patch lengths, which directly control the center frequency of each port. As illustrated in Fig. 7 (Fig. 7a and d), increasing the patch lengths (*l*_1_, *l*_2_, *l*_3_, and *l*_4_) shifts their corresponding resonant frequencies (*f*_1_, *f*_2_, *f*_3_, and *f*_4_) to lower bands. The achievable tuning ranges for each patch and their possible applications (beyond 5G/6G/IoT) are summarized in Table 4. This highlights that the proposed model is able to operate at any desired frequency across the entire 3.5–8.4 GHz range to cover any desired applications. It may be noted that, throughout the process, the patch width is kept constant; variations in width (*w*) cause slight changes in resonance depth and radiation characteristics, thereby offering a means for fine performance optimization.

**Fig. 8:**
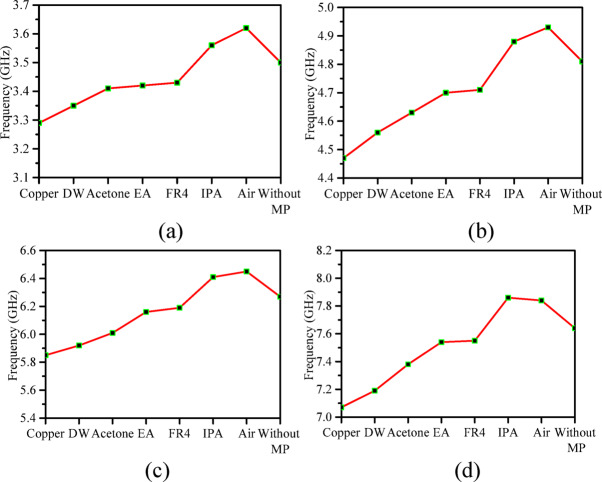
Resonating frequency response of each port of the RSIW backed self-quadruplexing antenna (**a**)-(**d**) without MP (micro-pockets) and with micro-pockets filled with various material like; Copper, Distilled Water (DW), Acetone, Ethyl Acetate (EA), Isopropyl alcohol (IPA), FR4, and Air.

**Table 4:** Prefabrication frequency scaling range and possible applications.

Parameter Range (mm)	Frequency (GHz)	Applications
10 ≤ *l*_1_ ≤ 13	3.5 ≤ *f*_1_ ≤ 4.42	Wi-Fi/Satellite Communication, IoT
7.8 ≤ *l*_2_ ≤ 8.9	4.81 ≤ *f*_2_ ≤ 5.35	Wi-Fi, ISM, RADAR, 5G/6G
5.4 ≤ *l*_3_ ≤ 6	6.27 ≤ *f*_3_ ≤ 6.72	Satellite Communication, RADAR, Wi-Fi 6E
3.4 ≤ *l*_4_ ≤ 4	5.66 ≤ *f*_4_ ≤ 8.4	Synthetic Aperture Radar (SAR), ISM, X-Band Radar Systems

**Table 5:** Frequency tuning based on different materials.

Materials	Frequency (GHz)			
	f_1_ (S/M)	f_2_ (S/M)	f_3_(S/M)	f_4_(S/M)
Copper	3.3/3.29	4.49/4.47	5.87/5.85	7.11/7.07
Distilled Water	3.5/3.35	4.61/4.56	5.95/5.92	7.2/7.19
Acetone	3.49/3.41	4.68/4.63	6.1/6.01	7.4/7.38
Ethyl Acetate	3.46/3.42	4.74/4.7	6.23/6.16	7.61/7.54
FR4	3.48/3.43	4.77/4.71	6.21/6.19	7.57/7.55
Isopropyl Alcohol	3.6/3.56	4.91/4.86	6.47/6.41	7.88/7.86
Air	3.71/3.62	4.98/4.93	6.49/6.45	7.89/7.84
Without Pocket	3.55/3.5	4.83/4.81	6.29/6.27	7.68/7.64

*S* Simulated, *M* Measured.

### Post-fabrication frequency tuning

Post fabrication tuning helps expand the frequency agility and offers an active frequency switch. Conventionally, this process includes diode-based tuning methods, embedding of tunable materials^15^. In diode-based tuning, by applying bias voltage to a PIN or Varactor diode, the frequencies are switched^15^. However, this technique is limited to a narrow tuning range and induces losses and increases design complexity^15^.

To overcome this limitation, a set of micro-cylindrical pockets are incorporated below the radiating patches, which can be filled with different materials (mostly in liquid form) via micro-pipette or syringe-based systems. This micro-loading changes the effective permittivity and the capacitance as well. This process directly enables effective tuning of the center frequencies at respective ports. This technique is suitable for compact, adaptive systems such as 5G/IoT sensor.

**Fig. 9:**
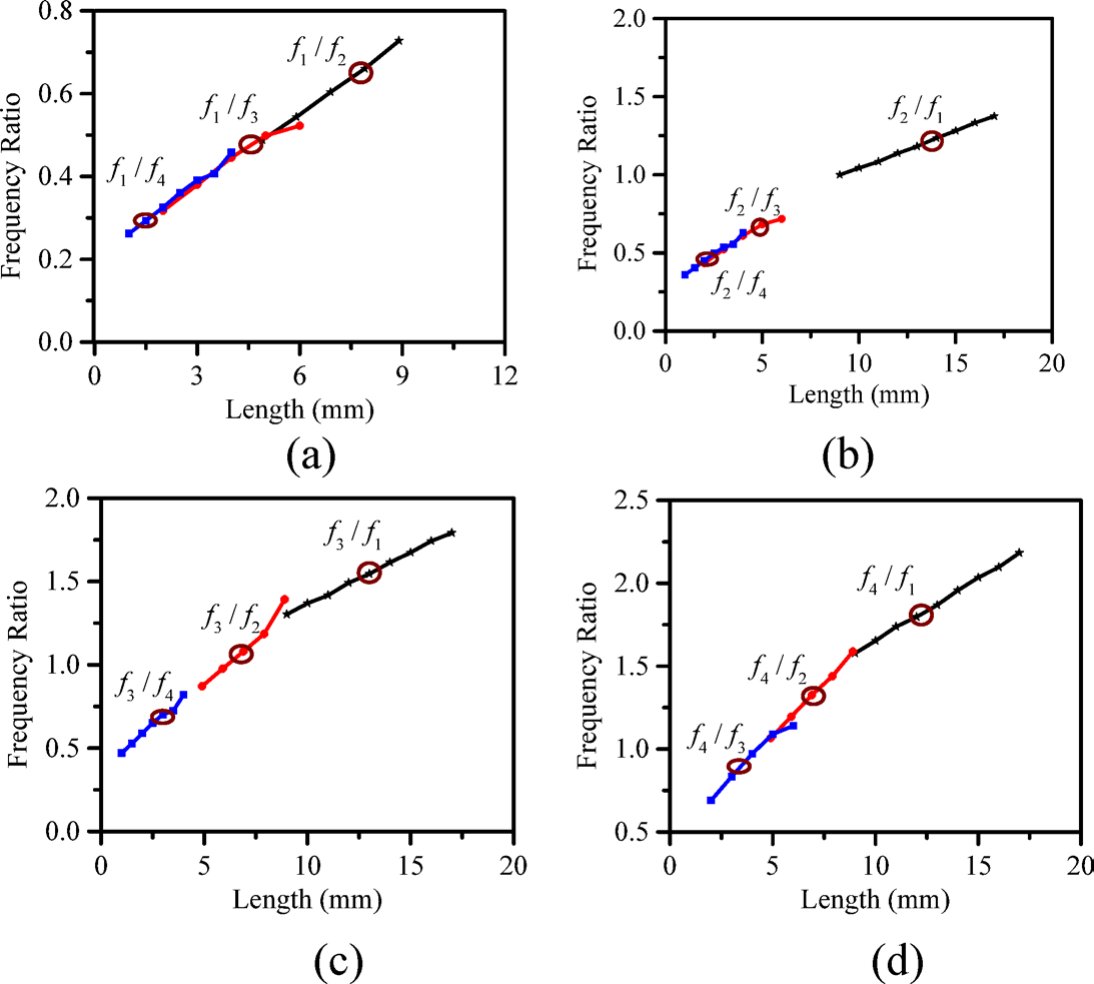
Frequency ratio (FR) for each frequency with respect to other frequencies (**a**) FR of *f*_1_ with *f*_2_, *f*_3_, and *f*_4_. (**b**) FR of *f*_2_ with *f*_1_, *f*_3_, and *f*_4_. (**c**) FR of *f*_3_ with *f*_1_, *f*_2_, and *f*_4_. (**d**) FR of *f*_4_ with *f*_1_, *f*_2_, and *f*_3_.

nodes, where continuous frequency tuning is required but difficult to achieve with conventional PIN diode designs. The frequency responses of the proposed prototype without micro-pockets and with micro-pockets filled with various materials are given in Fig. 8a-d. The frequency shift based on various materials is shown in Table 5. It may be noted that, extreme temperature variation may slightly affect the dielectric property of the liquid but the antenna operation remains stable.

### Frequency ratio

Ensuring closely spaced resonant frequencies with high isolation and tuning independence in a multi-port antenna is a big challenge. To understand this frequency ratio (FR) of each resonating frequency with its adjacent frequency w.r.t. corresponding patch length (*l*_1_/*l*_2_/*l*_3_/*l*_4_) is calculated and shown in Fig. 9. It reads that the FR for each case varies monotonically w.r.t. respective patch lengths, which indicates independent frequency tuning and stable multiplexing characteristics. It also shows that the antenna is capable of operating at very closely spaced frequencies and is suitable for systems that require compact and precise frequency allocation across adjacent bands.

### Prototype and result validation

**Fig. 10:**
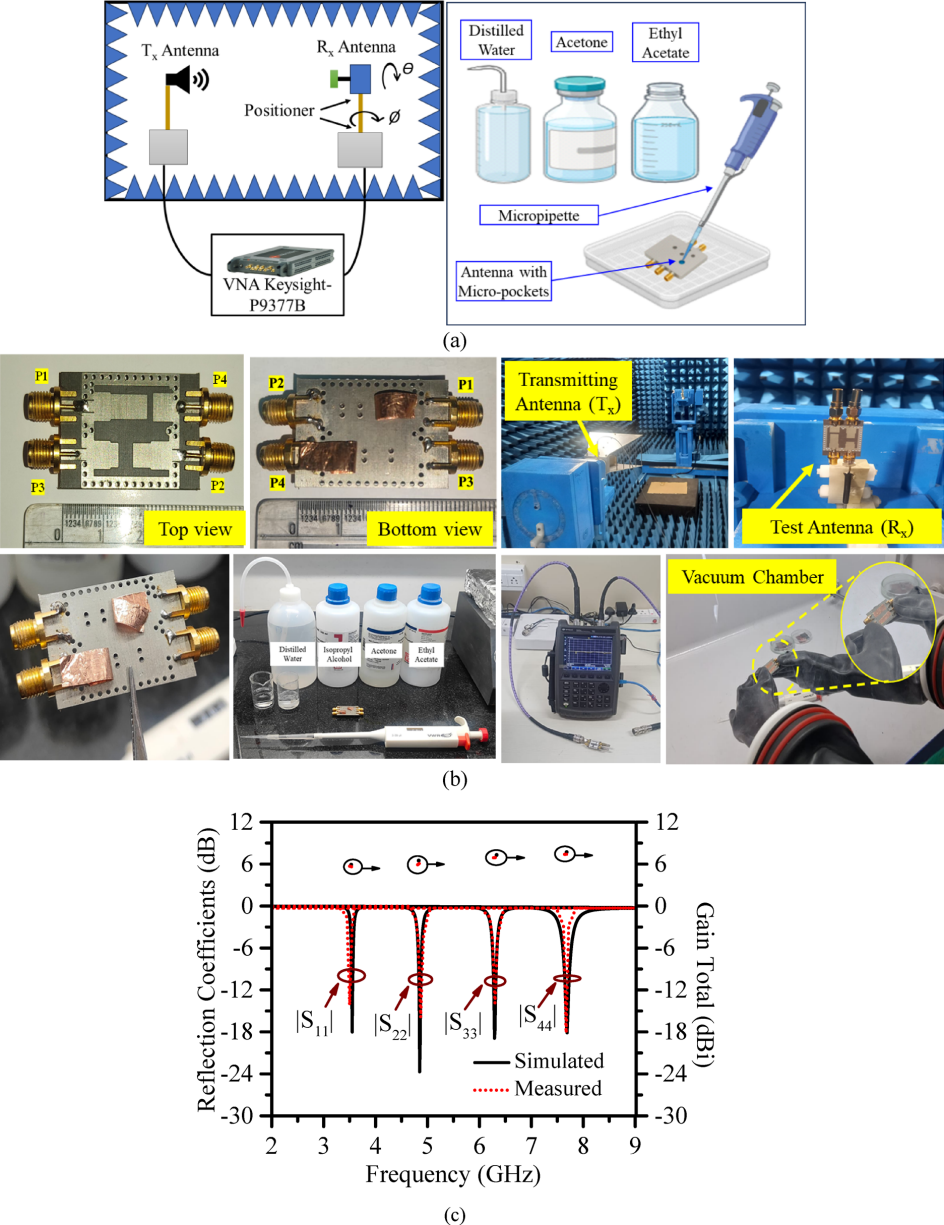

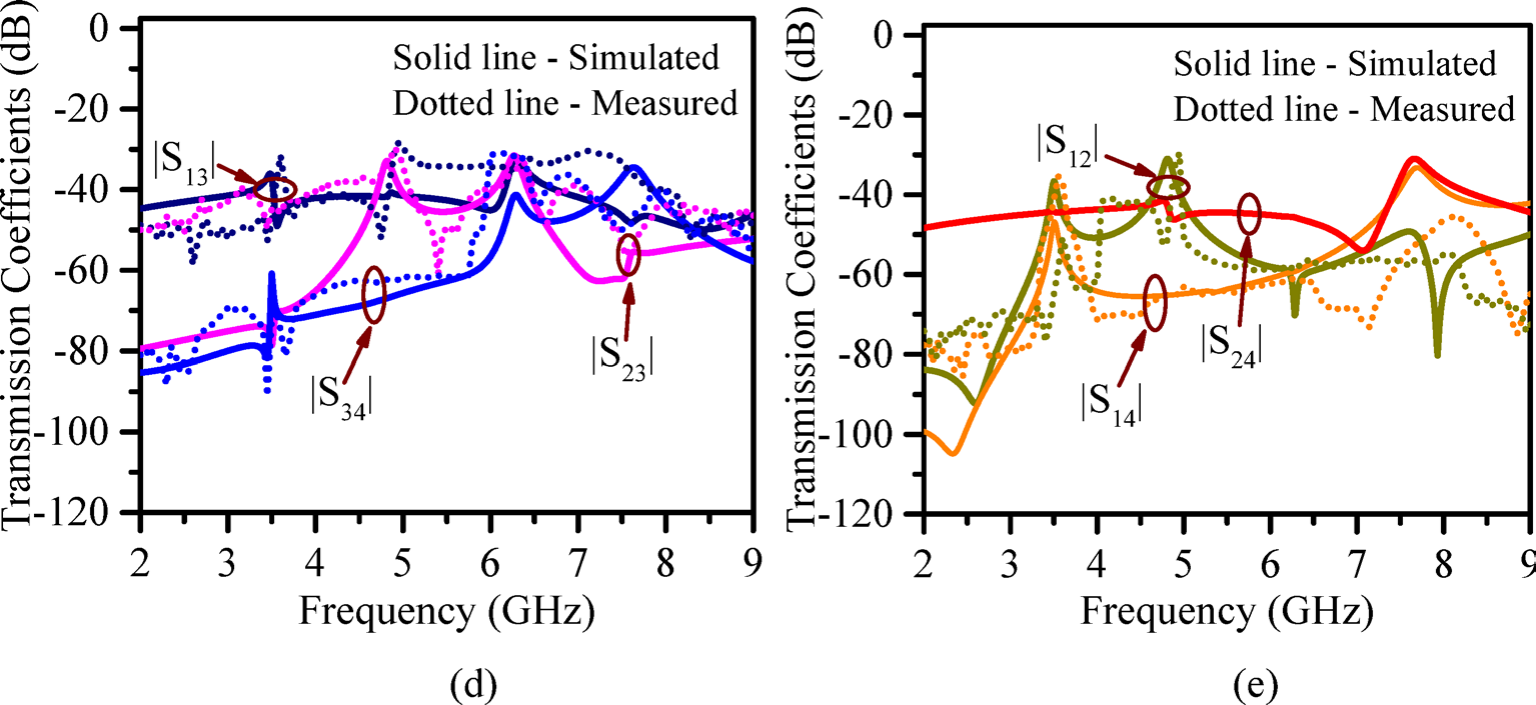
(**a**) Antenna measurement setup sketch, (**b**) Actual measurement setup inside lab with fabricated prototype, (**c**), (**d**), (**e**) Comparison of simulated and measured reflection coefficient and gain total, transmission coefficients, respectively.

The proposed model is prototyped on RT/Duroid 5870 substrate (*ε*_*r*_ = 2.33, *tan δ* = 0.0012 and height = 0.787 mm) and analyzed for further validation. Standard PCB fabrication technique and through-drill vias were used to realize the proposed RSIW cavity. Sketch of measurement setup showing the vector network analyzer and anechoic-chamber arrangement and the liquid-filling process in micro-pockets is shown in Fig. 10a. It may be noted that the micro-pockets (height = 0.5 mm and diameter = 1 mm) are filled carefully using micropipette and post filling, the pockets are sealed using adhesive conducting tapes. This entire process was done inside a vacuum box as shown in Fig. 10b, which ensures no bubble, no leakage, and maintain ground plane continuity. Figure 10b illustrates the fabricated antenna prototype in a standalone condition and during measurement, showing the anechoic chamber, vector network analyzer (VNA), and the setup for filling dielectric liquids into the micro-pockets. S-parameters are measured at each port, while the rest ports were terminated with a 50Ω matched load. The measured and simulated S-parameters without microfluidic pockets are compared in Fig. 10c-e. It shows excellent reflection coefficient matching with acceptable deviations or offsets in transmission coefficients. The observed minimum isolation is better than 32 dB. The radiation characteristics are tested inside an anechoic chamber. The antenna peak gain is measured at 7.6 dBi.

**Fig. 11:**
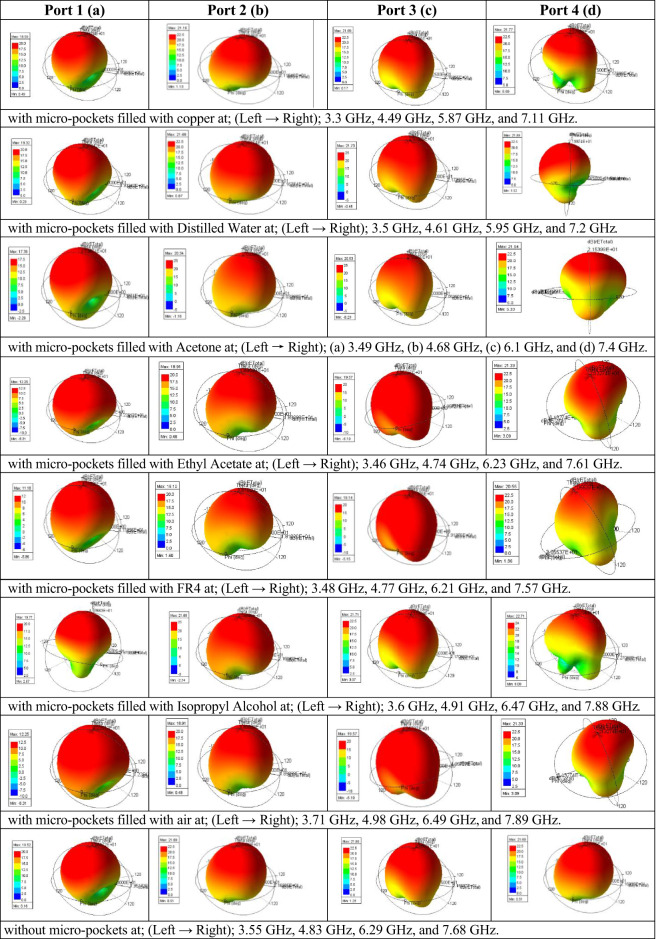
3D radiation pattern of the proposed antenna model w.r.t. different ports at different frequencies.

**Fig. 12:**
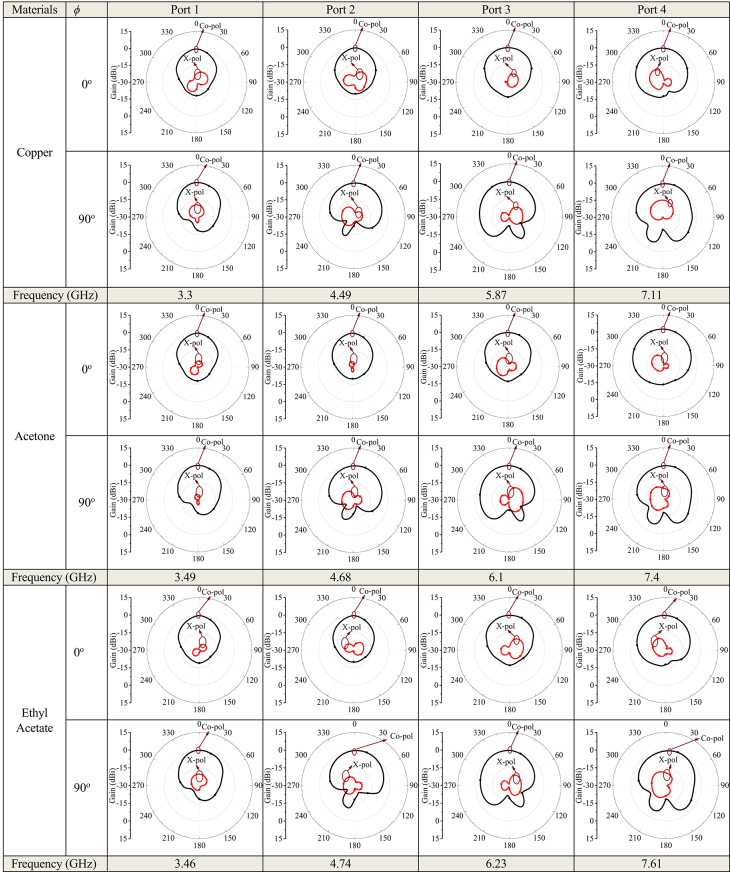

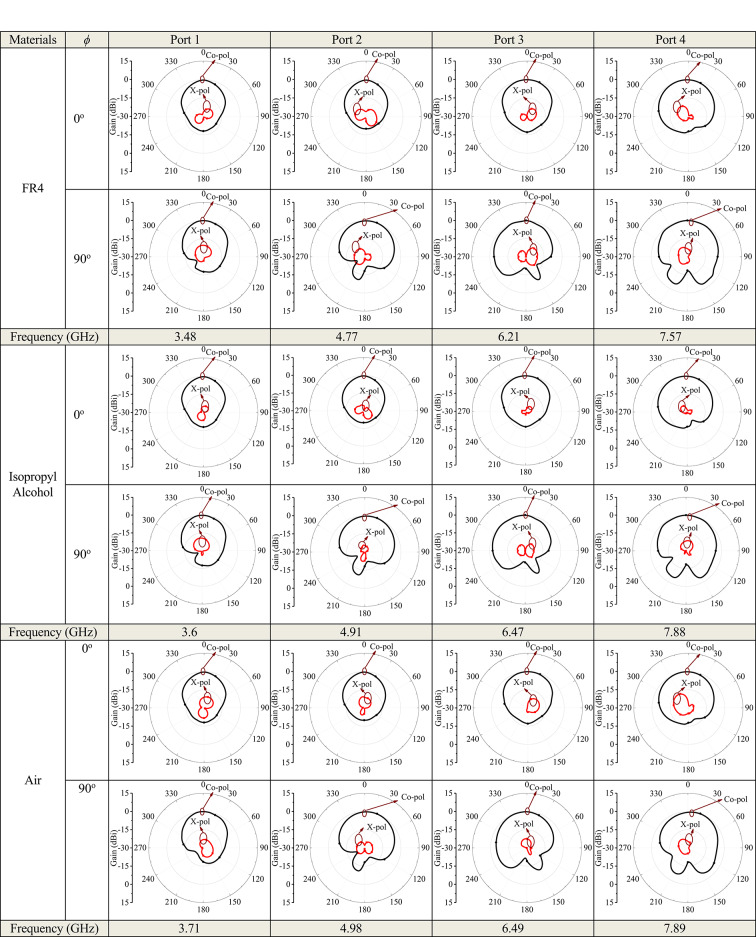

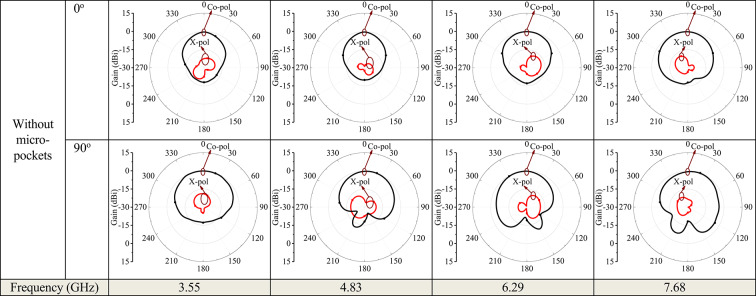
Simulated radiation pattern of the proposed quad band antenna structure with incorporation of micro-pockets filled with various materials – copper, acetone, ethyl acetate, Isopropyl alcohol, FR4, air and without micro-pockets; (Black – Co-pol, Red – X-pol).

**Fig. 13:**
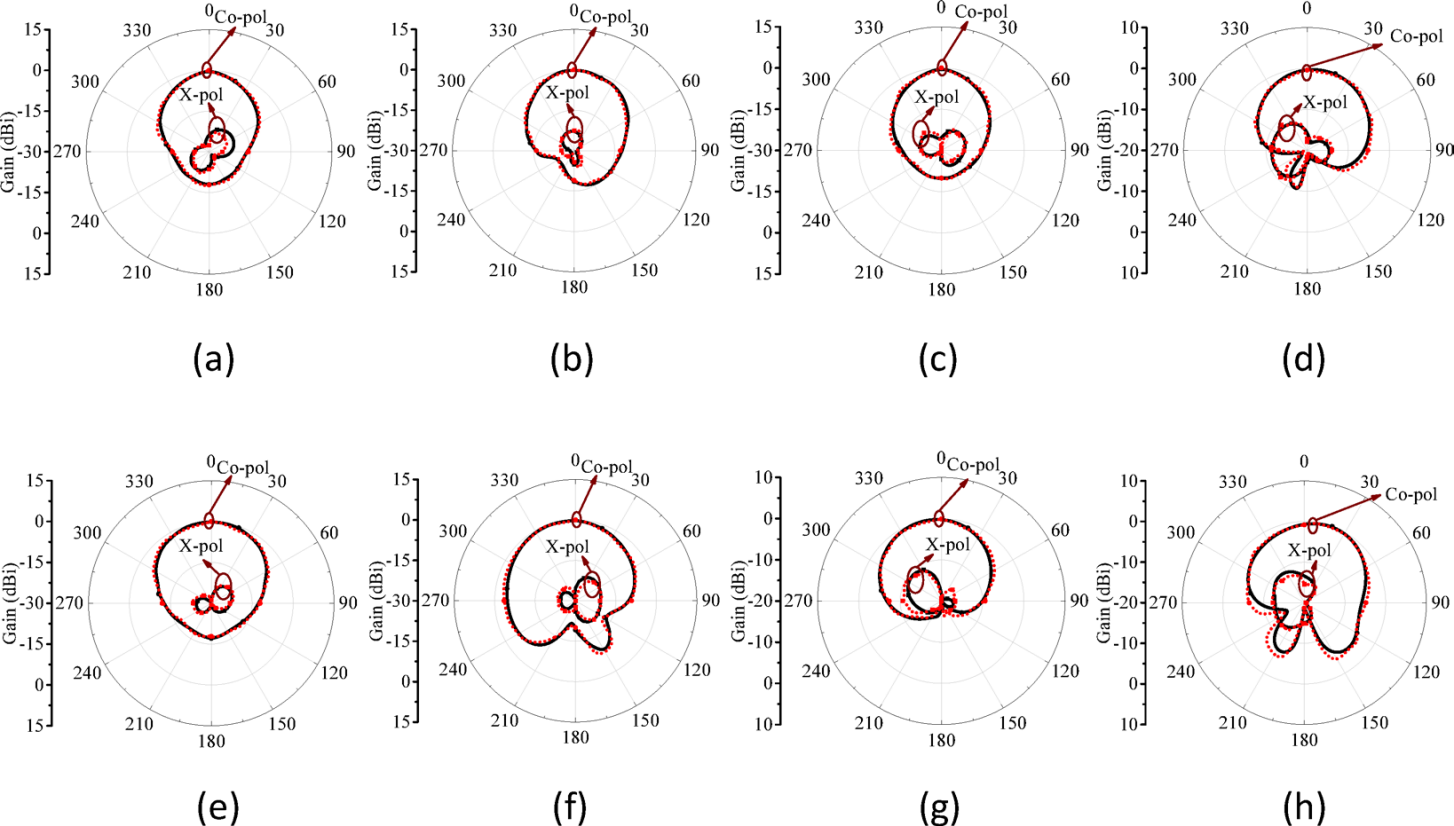
Simulated Vs Measured radiation pattern of the proposed antenna quad band structure with incorporation of micro-pockets filled with distilled water; (**a**), (**b**) 3.35 GHz, (**c**), (**d**) 4.56 GHz, (**e**), (**f**) 5.92 GHz, and (**g**), (**h**) 7.19 GHz. (Left - *ϕ* = 0^o^, Right - *ϕ* = 90^o^, Black – Simulated, Red – Measured).

Figure 11 illustrates the simulated 3D radiation patterns of the proposed antenna without micro-pockets and with micro-pockets filled with various materials, confirming broadside radiation characteristics, good directional stability, and minimal distortion. This also indicates proper aperture utilization and low sidelobe levels. Figure 12 shows the simulated 2D radiation pattern of the proposed model at all ports with micro-pockets filled with various materials for *ϕ* = 0^o^ and *ϕ* = 90^o^. It clears that, in all cases the co-polarizations and cross-polarizations are well separated with nearly no overlapping across all frequency bands. The overall co-pol to cross pol differences are 20 dB (min) and 27 dB (max) in broadside direction and observed FTBR is better than 17.83 dB. This confirmed the tuning of the antenna hardly affect the fundamental radiatin pattern. The measured radiation patterns at all resonating frequencies for the proposed model with micro-pockets filled with distilled water is depicted in Fig. 13. This shows the measured results well matches with the simulated ones except minimal to negligible variations.

The antenna demonstrates a co-polarized gain in the range of 5.91 dBi to 7.6 dBi across all operating bands. The cross-polarization level remains well suppressed with values better than 10 dB. Stable radiation characteristics are maintained consistently over the entire frequency range. The overall results show close agreement between simulated and measured data, validating the antenna’s quad-band performance.

**Fig. 14:**
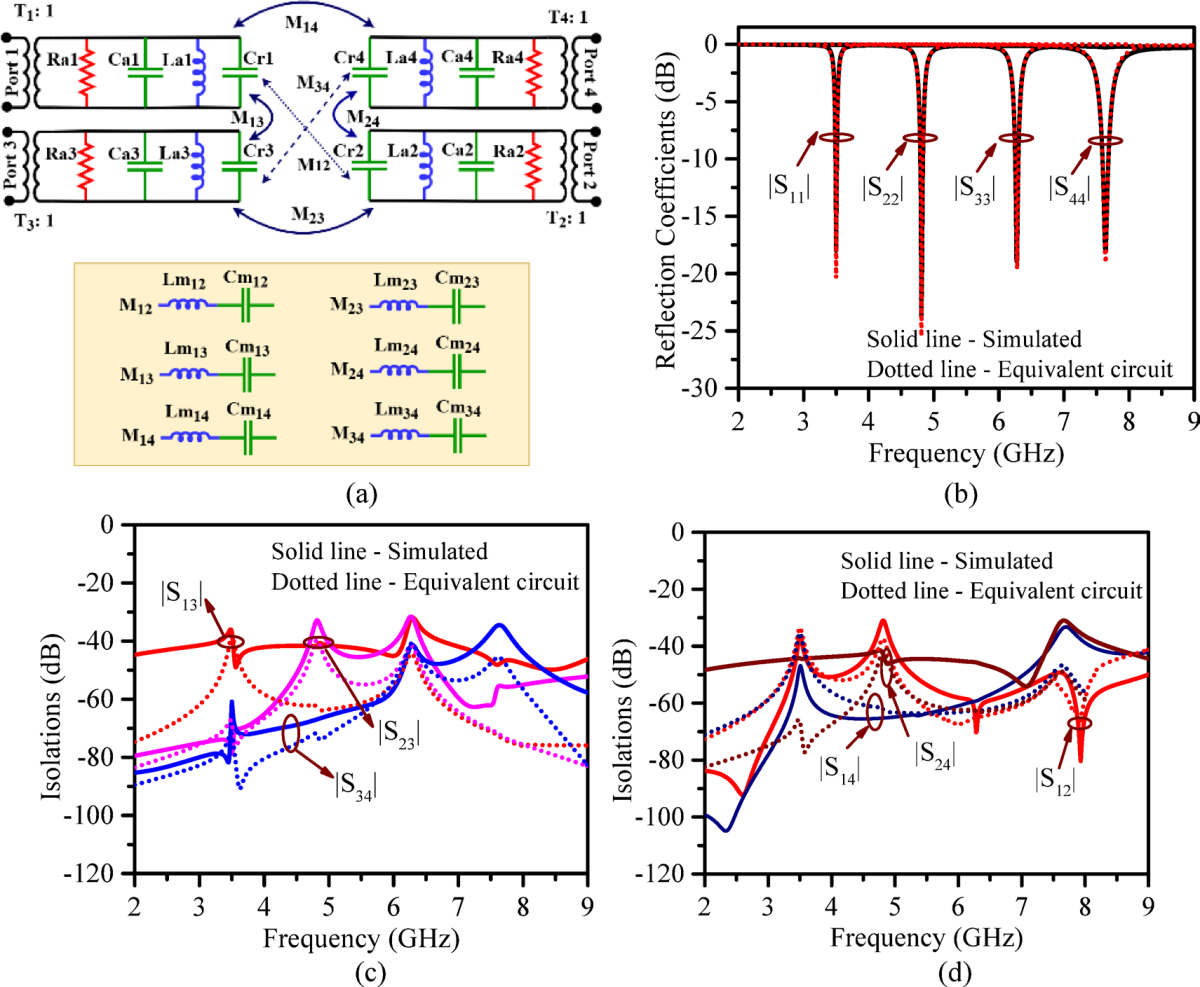
(**a**) Equivalent circuit representation of the proposed structure, comparison of HFSS simulations (EM model) versus ADS simulations (equivalent circuit); (**b**) reflection coefficients, (**c**), (**d**) isolations.

The equivalent circuit model of the proposed RSIW antenna is shown in Fig. 14a. The transition from microstrip feed line to radiating element is represented using a transformer model for impedance matching. The radiating patches are constructed as a parallel RLC circuit denoted commonly as *R*_*ax*_, *C*_*ax*_ and *L*_*ax*_ (*x* →1 to 4) and the slots associated with each port are added as shunt capacitance (*C*_*rx*_, where *x* →1 to 4) to the end of the RLC branch. The capacitive elements are inserted at the end to simulate the physical slot gaps/isolation. The inter port coupling denoted as *Mc*_*ij*_ where *i*,* j* → 1 to 4 and *j* > *i* is modelled by additional series LC pairs between the port circuits. The detailed circuit model component values are tabulated in Table 6. The simulation of the equivalent circuit is done using Keysight ADS 2019 and the obtained S- parameter curves are compared with the simulated S-parameter obtained from HFSS and are presented in Fig. 14b-d.

**Table 6:** Equivalent Circuit Component Specifications of the Proposed Antenna Structure Without Micro-Pockets.

Component	Value	Component	Value
*R*_*a*1_	930 Ω	*Lm*_12_	34.6 nH
*R*_*a*2_	700 Ω	*Lm*_13_	28.72 nH
*R*_*a*3_	640 Ω	*Lm*_14_	19.54 nH
*R*_*a*4_	490 Ω	*Lm*_23_	47.59 nH
*L*_*a*1_	0.546 nH	*Lm*_24_	44.677 nH
*L*_*a*2_	0.3 nH	*Lm*_34_	69 nH
*L*_*a*3_	0.185 nH	*Cm*_12_	6.546 pF
*L*_*a*4_	0.216 nH	*Cm*_13_	6.861 pF
*C*_*a*1_	2.775 pF	*Cm*_14_	7.56 pF
*C*_*a*2_	2.123 pF	*Cm*_23_	9.578 pF
*C*_*a*3_	1.682 pF	*Cm*_24_	7.236 pF
*C*_*a*4_	0.978 pF	*Cm*_34_	8.67 pF
*C*_*r*1_	1.248 pF	T_*1*_	0.211
*C*_*r*2_	1.601 pF	T_*2*_	0.269
*C*_*r*3_	1.838 pF	T_*3*_	0.309
*C*_*r*4_	1.071 pF	T_*4*_	0.286

**Table 7:** Comparison of the proposed work with other existing designs.

Ref/Year	Frequency (GHz)	Band	Size (λ_g_²)	Gain (dBi)	ISL (dB)	FTBR (dB)	Pol. Dt.	Pr. FT	Po. FT	Circuit Model	Tuning Methods
^8^2023	3.5–3.8, 5.53–6.2	Dual	0.22	5.04–5.58, 5.26–5.96	> 27	> 19.4	Y/Y	Yes	Yes	Yes	Distilled Water filled Micro-pockets
^9^ 2024	2.7–3.47, 4.05–4.96	Dual	0.31	1.97–4.43, 3.66–4.95	> 20.35	N.A.	Y/Y	Yes	Yes	Yes	Solid and liquid dielectric filled micro-pockets
^10^ 2024	4.70–5.23, 5.55–6.34	Dual	0.11	> 4.7, > 5.5	> 23.5	N.A.	Z/Z	Yes	Yes	Yes	By varying slot length and inserting DR of different permittivity
^11^ 2025	3.5, 4.9	Dual	0.25	4.38, 5.98	> 22.89	N.A.	Z/Z/Z/Z	Yes	No	No	By varying slot dimensions
^13^ 2018	4.18, 5.2, 5.8	Tri	1.04	6.56, 4.2, 5.85	> 23	> 19	Z/Y/Y	Yes	No	No	By varying slot length
^14^ 2020	4.14, 6.1, 8.32	Tri	0.17	4.26, 4.41, 6.27	> 30.8	> 15	X/X/Y	Yes	No	No	By varying patch length
^15^ 2024	4.8, 5.8, 6.6	Tri	0.18	5.9, 6.2, 6.3	> 22	N.A.	Z/Z/Z	Yes	No	Yes	By varying slot length and dielectric constant
^16^ 2025	5.25, 28.85, 31.9	Tri	0.49	5.1, 8.1, 8.3	> 33	N.A.	X/Y/X	Yes	No	Yes	By varying slot length
^17^ 2019	8.19, 8.78, 9.71, 11	Quad	0.77	5.5, 6.9, 7.47, 7.45	> 22	> 18.2	Y/X/Y/X	Yes	No	No	By varying slot length
^18^ 2020	5.14, 5.78, 6.74,7.74	Quad	0.27	4.1, 4.96, 6.2, 6.1	> 28	> 17.5	X/X/Y/Y	Yes	No	No	By varying resonating patch’s length.
^19^ 2020	8.85, 10.4, 11.4, 12.23	Quad	1.369	5.2, 6, 6.25, 7	> 27	> 15	X/Y/Y/X	Yes	No	No	By varying stub length
^20^ 2022	4.8, 5.4, 28, 30	Quad	0.51	5.4, 5.2, 8, 8.7	> 20	N. A.	Y/Y/X/X	Yes	No	No	By varying slot length and by adding vias and changing its position.
^21^ 2023	2.33, 2.96, 5.43, 6.15	Quad	0.125	4.21, 3.39, 6.1, 4.34	> 32.5	> 8	Y/Y/X/X	Yes	No	Yes	By varying patch length
^22^ 2023	5.8, 7.4, 28, 38	Quad	0.25	4.1, 5.2, 6.1, 8.3	> 26	N.A.	X/Y/Y/X	Yes	No	Yes	By varying slot length
^23^ 2024	4.07, 4.65, 5.45, 6.20	Quad	0.192	4.29, 4.24, 4.05, 3.81	> 27	> 14.17	X/X/Y/Y	Yes	Yes	Yes	Air and distilled water filled micro-pockets
^24^ 2024	5.17, 5.76, 3.55, 7.03	Quad	0.27	5.83, 5.68, 6.71, 6.67	> 23.5	N.A.	X/X/Y/Y	Yes	Yes	Yes	Micro-pockets filled with various materials
^25^ 2024	4.22, 4.85, 5.45, 5.98	Quad	0.37	4.85, 4.82, 4.40, 4.05	> 32.3	> 15.02	X/Y/X/Y	Yes	Yes	Yes	Micro-fluidic pockets
^26^ 2025	4.3, 5.2, 5.8, 6.9	Quad	0.11	5.19, 5.63, 6.88, 7.57	> 29.1	N.A.	Z/Z/Z/Z	Yes	No	Yes	By varying slot length
TW 2025	3.29, 4.47, 5.85,7.07	Quad	0.09	5.91, 6.3, 7.18, 7.6	> 32	> 17.83	X/X/X/X	Yes	Yes	Yes	Varying patch lengths and Micro-pockets filled with various materials

*TW* This work, *ISL* Isolation, *FTBR* Front-To-Back-Ratio, *Pol. Dt.* Polarization Direction, *X* X-axis, *Y* Y-axis, *Z* Z-axis, *Pr. FT* Pre-fabrication Tuning, *Po. FT* Post-fabrication Tuning, *DR* Dielectric Rods, *N.A.* Not Available.

The performance metrics of the proposed work are compared with the existing model of a similar class and reported in Table 7. The key observations from the table are;

i) Most of the reported models uses either varying slot/stub length^10–22^ or micro-pocket techniques^8,9^,^23–25^ for frequency tuning. In contract, the proposed model supports both techniques for tuning over wide frequency range.
ii) The proposed model is 18.18% more compact than the most compact model reported in^26^ with a peak gain of 7.6 dBi.
iii) It has achieved a front-to-back ratio (FTBR) > 17.83 dB, higher than those reported in^14^^,19^^,21^^,23^^,25^ and with an improvement of 18.7% over^25^.
iv) The proposed model also obtained a strong isolation (32 dB) compared to^8–15^,^17–20^,^22–24^^,26^.
v) The reported literature includes designs with different polarizations making them unfit to be considered as a true multiband alternative. Interestingly, this model offers single polarization combined with post-fabrication tuning capability making it a true multiband alternative.

Looking at the future perspective and sustainability, the proposed micro-pocket-based frequency reconfiguration technology can be extended to higher-order self-multiplexing antennas as well as MIMO antennas.

## Conclusion

A compact single-polarized, substrate integrated cavity-based self-quadruplexing antenna resonating at four different frequencies has been designed and validated. The proposed antenna is excited by four 50-ohm microstrip feedlines, which enables easy integration with planar devices. The prototype achieved resonant frequencies at 3.29 GHz, 4.47 GHz, 5.85 GHz, and 7.07 GHz with isolation better than 32 dB, along with a FTBR of more than 17.83 dB and a peak gain of 7.6 dBi. The measured gains of the proposed prototype without the incorporation of micro-pockets are 5.91 dBi at 3.5 GHz, 6.3 dBi at 4.81 GHz, 7.18 dBi at 6.27 GHz, and 7.6 dBi at 7.64 GHz, confirming consistent radiation performance across all four bands. The frequency agility of the proposed model is achieved using both pre-fabrication and post-fabrication tuning methods. Before fabrication, tuning from 3.5 GHz to 8.4 GHz has been achieved by modifying the patch length. After fabrication, the prototype can be tuned from 3.29 GHz to 7.84 GHz by incorporating micro-pockets filled with different materials, allowing the antenna to be reconfigured without requiring additional fabrication, thereby reducing the production cost and time. The mentioned frequency agile techniques enable the antenna to adapt or be reconfigured to a wide range of application requirements. When compared with existing state-of-the-art designs, the proposed model is more compact with an area of 0.09*λ*_*g*_² and offers single polarization with post-fabrication tuning capability, making it suitable for future high-performance communication applications, including but not limited to 5G/6G IoT devices.

## Data Availability

The datasets used and/or analyzed during the current study are available from the corresponding author on reasonable request.
